# Lower Extremity Kinematic and Kinetic Adaptations During a Half Marathon

**DOI:** 10.1002/ejsc.70069

**Published:** 2025-10-13

**Authors:** Wenjin Wang, Shulin Xu, Igor Komnik, Josef Viellehner, Jingmin Liu, Wolfgang Potthast

**Affiliations:** ^1^ Institute of Biomechanics and Orthopedics German Sport University Cologne Cologne Germany; ^2^ Department of Sports Science and Physical Education Tsinghua University Beijing China

**Keywords:** half‐marathon, kinematics, kinetics, symmetry angle

## Abstract

This study aimed to clarify the biomechanical adaptations recreational runners experience during a half marathon. Thirty‐seven healthy runners completed a half marathon as fast as possible on a laboratory‐instrumented treadmill. Kinematic and kinetic data were collected every 5 km from 0 to 20 km. As mileage accumulated, peak vertical ground reaction force (GRF) decreased from 5 km onward (*p* < 0.005) and impulse declined from 10 km (*p* < 0.05). Ground contact time increased from 10 km (*p* < 0.05). Joint‐level adaptations included an increased hip extension moment (*p* < 0.005) and hip adduction angle from 10 km (*p* < 0.05), decreased knee abduction moment from 5 km (*p* < 0.005), and greater knee flexion angle at 10 km (*p* < 0.001) and 15 km (*p* = 0.031). Additionally, the symmetry angle of peak vertical GRF decreased at 20 km, suggesting improved interlimb symmetry under fatigue. These findings provide insight into fatigue‐related biomechanical adaptations during long‐distance running. Further research is needed to clarify how these changes relate to injury development.

## Highlights

- Peak vertical ground reaction force decreased from 5 km onward, whereas ground contact time increased from 10 km as mileage accumulated during a half marathon on treadmill.
- Joint‐level adjustments included increased hip extension moment and hip adduction angle from 10 km and decreased knee abduction moment from 5 km.
- The symmetry angle of peak vertical ground reaction force decreased at 20 km, suggesting improved interlimb symmetry under fatigue.

## 1 Introduction

Participation in marathons has grown over the past decade, with global race data showing a 49% rise in participation from 2008 to 2018 (Andersen and Nikolova 2019). However, running‐related musculoskeletal injuries remain a concern, with a reported prevalence of 44.6% ± 18.4% (Kakouris et al. 2021). Fatigue from prolonged running often leads to altered movement patterns, which can heighten the risk of injury (Gijon‐Nogueron and Fernandez‐Villarejo 2015). Understanding how the accumulation of mileage during a half marathon impacts landing strategies, joint kinematics, and kinetics is crucial to identifying potential mechanisms of injury in the lower extremities.

A recent systematic review emphasized that biomechanical risk factors for running‐related injuries are often injury‐specific and frequently involve joint mechanics (Willwacher et al. 2022). Among these, ground reaction force (GRF) and ground contact time are key determinants of the impulse generated during running. Previous studies have shown that fatigue increases contact time while reducing GRF and impulse (Apte et al. 2021). However, how these parameters evolve throughout a half marathon remains unclear. Additionally, fatigue‐related changes in lower extremity kinematics can affect running gait as demonstrated in prolonged running tasks (Wang et al. 2024). Patellofemoral pain, a common running injury, affects 17% of runners and is associated with distinctive biomechanical patterns (Ceyssens et al. 2019; Neal et al. 2016). It is still uncertain how variations in joint angles and moments during a half marathon may contribute to the risk of developing patellofemoral pain.

Gait asymmetry reflects a natural functional difference between the limbs and may relate to the specific contributions of each limb to propulsion and control tasks (Sadeghi et al. 2000). Recent prospective evidence in a large cohort of recreational runners indicates that gait asymmetry is not associated with a higher risk of running‐related injury (Malisoux et al. 2024). However, asymmetry affects running efficiency, a 10% increase in asymmetry in ground contact time and GRF has been shown to raise metabolic costs by 7.8% and 3.5%, respectively (Beck et al. 2018). Although fatigue‐induced asymmetry has been observed in hip and knee kinematics and kinetics (Gao et al. 2022), how these asymmetries evolve during a half marathon remains unclear.

This study focuses on peak vertical GRF, ground contact time, impulse, joint angles and moments, and lower extremity asymmetry. Ground contact time and impulse reflect changes in external loading and propulsion strategies; peak vertical GRF is mainly related to propulsion; joint angles and moments provide insight into neuromuscular control and compensatory mechanisms (Apte et al. 2021); and asymmetry has been linked to running efficiency (Beck et al. 2018; Guan et al. 2022). Therefore, the aim of this study was to investigate the effects of accumulated running mileage during a half marathon on these biomechanical parameters. Based on previous research (Gao et al. 2022; Chen et al. 2022; Wang, Zhang, et al. 2025), we hypothesized that as the half marathon progresses, there would be a reduction in peak vertical GRF and impulse, an increase in ground contact time, significant alterations in joint kinematics and kinetics, and increased lower extremity asymmetry.

## 2 Methods

### 2.1 Participants

A sample size calculation was performed for the main variable of interest, for example, peak vertical GRF. Using G*Power, and assuming an effect size of 0.25, a significance level of 0.05, and a power of 0.80, the required number of participants is 22. We recruited 37 recreational runners (mean age: 28.4 ± 5.6 years; body mass: 64.3 ± 10.5 kg; and height: 1.72 ± 0.08 m), including 20 females (mean age: 26.0 ± 4.3 years; body mass: 59.2 ± 9.2 kg; and height: 1.67 ± 0.07 m) and 17 males (mean age: 31.1 ± 5.8 years; body mass: 70.4 ± 8.7 kg; and height: 1.77 ± 0.06 m), from a local running club and our university using posters. Inclusion criteria included were as follows: age 20–40 years, completion of at least one half marathon in the past 2 years, regular running (1–3 times per week for at least 6 months), a minimum weekly distance running of 20 km, rear‐foot striking gait, and no significant injuries in the past 6 months. Participants primarily ran for health or leisure. The study was approved by the Institutional Review Board of the German Sport University Cologne (No. 141/2022), and all participants provided written informed consent.

### 2.2 Experiment Protocol

**FIGURE 1:**
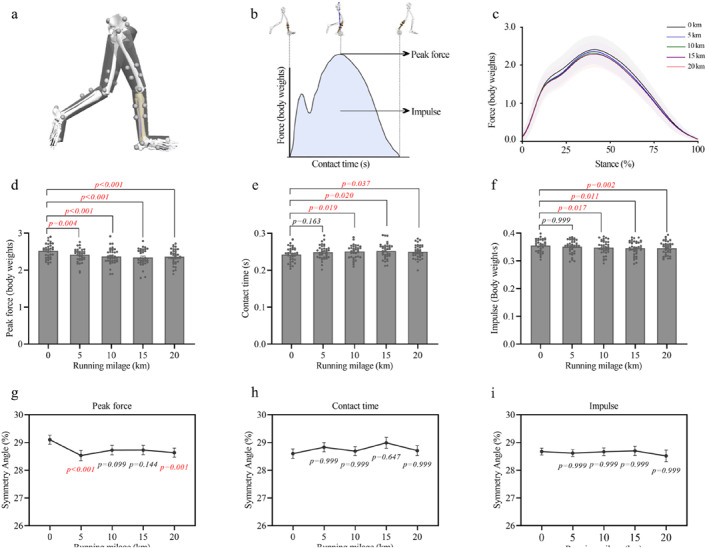
Changes in ground reaction force, contact time, and impulse, along with their symmetry angles during the half marathon. (a) Marker placement for motion capture. (b) Illustration of contact time, peak GRF, and impulse. (c) Average vertical GRF waveform across participants (bold line = mean and shaded area = SD). (d–f) Mean ± SEM values of peak vertical GRF (d), contact time (e), and impulse (f) across running distances. (g–i) Mean ± SEM of symmetry angles for peak vertical GRF (g), contact time (h), and impulse (i) during the stance phase.

All participants had prior treadmill experience and completed a 5‐min familiarization run at a self‐selected pace before data collection. Reflective markers were placed on specific bony landmarks, covering seven anatomical segments as follows: the pelvis, bilateral thighs, shanks, and feet (Figure 1a). Participants ran a half marathon on a laboratory‐instrumented treadmill, aiming to complete the distance as fast as possible at maximum effort, with self‐selected pace adjustments allowed throughout the run (mean time: 2:01:06 ± 0:11:47). Kinematic and kinetic data were synchronously collected at 10 km/h for 20 s at five intervals as follows: at 0, 5, 10, 15, and 20 km. Data collection used an eight‐camera Oqus system (200 Hz; Qualisys, Sweden) and an instrumented treadmill (2000 Hz; H/p cosmos, Germany).

### 2.3 Data Analysis

Marker trajectories were processed using Qualisys Track Manager for labeling and gap‐filling. Data were then imported into Visual3D (C‐Motion, Rockville, MD, USA), low‐pass filtered using a fourth‐order Butterworth filter with a cutoff frequency of 20 Hz. A lower extremity model was used to calculate peak knee and hip angles (°), peak knee and hip moments (Nm/kg), peak GRF (N/BW), impulse (N·s/BW), and contact time (s) during the stance phase of running. The stance phase was identified using vertical GRF data, defined from initial ground contact to foot‐off (20 N threshold). Average peak values from 10 consecutive gait cycles were used for analysis. Asymmetry was calculated using the symmetry angle (Zifchock et al. 2008):

$$\text{Symmetry}\;\text{angle}=\left(45{}^{\circ}-\arctan\left(X_{\text{left}}\;/\;X_{\text{right}}\right)\right)\;/\;90{}^{\circ}\times 100\%.$$

If 45° − arctan (*X*_left_/*X*_right_) > 90°, then a substitution was applied (Exell et al. 2017):

$$\text{Symmetry}\;\text{angle}=\left(\left(45{}^{\circ}-\arctan\left(X_{\text{left}}\;/\;X_{\text{right}}\right)-180{}^{\circ}\right)\;/\;90{}^{\circ}\right)\times 100\%.$$

### 2.4 Statistical Analyses

Statistical analyses were conducted using SPSS software (IBM Corp., US) and Prism v.9.0 (GraphPad). Data normality was assessed with the Shapiro–Wilk test. Repeated‐measures ANOVA with a Bonferroni correction for multiple comparisons was used. The significance level was set at *p* < 0.05.

## 3 Results

### 3.1 GRF, Impulse, and Contact Time

Mileage accumulation influenced peak vertical GRF, impulse, and contact time during the stance phase of running (Figure 1). Peak vertical GRF (Figure 1d) decreased significantly over distance, with a main effect of time (*p* < 0.001 and *η* ^2^_p_ = 0.590). It declined from 2.52 ± 0.04 N/BW at 0 km to 2.42 ± 0.03 N/BW at 5 km (−3.89% ± 1.01% and *p* = 0.004), 2.37 ± 0.04 N/BW at 10 km (−5.79% ± 1.03% and *p* < 0.001), 2.34 ± 0.04 N/BW at 15 km (−6.89% ± 1.08% and *p* < 0.001), and 2.36 ± 0.03 N/BW at 20 km (−6.12% ± 0.99% and *p* < 0.001). Impulse (Figure 1f) also showed a significant decrease (*p* = 0.001 and *η* ^2^_p_ = 0.416), decreasing from 0.355 ± 0.004 N·s/BW at 0 km to 0.348 ± 0.004 N·s/BW at 10 km (−2.02% ± 0.60% and *p* = 0.017), 0.346 ± 0.004 N·s/BW at 15 km (−2.56% ± 0.73% and *p* = 0.011), and 0.346 ± 0.004 N·s/BW at 20 km (−2.64% ± 0.65% and *p* = 0.002). In contrast, contact time increased significantly with distance (*p* = 0.029 and *η* ^2^_p_ = 0.279), rising from 0.242 ± 0.003 s at 0 km to 0.251 ± 0.003 s at 10 km (+3.67% ± 1.12% and *p* = 0.019), 0.252 ± 0.003 s at 15 km (+4.22% ± 1.22% and *p* = 0.020), and 0.250 ± 0.003 s at 20 km (+3.30% ± 1.01% and *p* = 0.037).

The symmetry angle of peak force (Figure 1g) decreased significantly from 29.10% ± 0.16% at 0 km to 28.53% ± 0.19% at 5 km (−1.95% ± 0.41% points and *p* < 0.001) and to 28.73% ± 0.17% at 20 km (−1.59% ± 0.38% points and *p* = 0.001). No significant changes were observed in the symmetry angles of contact time (Figure 1h) and impulse (Figure 1i) across the run.

### 3.2 Hip Joint

**FIGURE 2:**
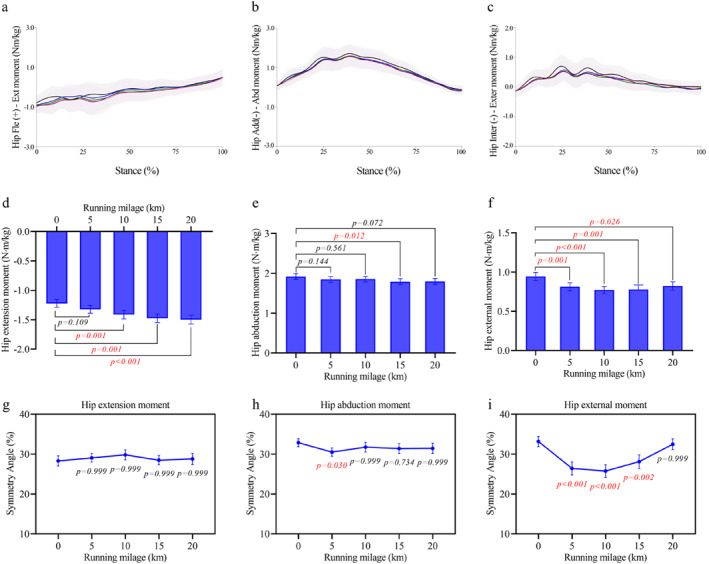
Changes in hip joint moments and their symmetry angles during the half marathon. (a–c) Average moment curves for hip extension (a), hip abduction (b), and hip external rotation (c) throughout the stance phase. Shaded areas indicate standard deviation. (d–f) Mean ± SEM values of peak hip extension moment (d), hip abduction moment (e), and hip external rotation moment (f) across running distances. (g–i) Mean ± SEM of symmetry angles for hip extension (g), hip abduction (h), and hip external rotation (i) moments.

Accumulated mileage affected hip joint mechanics. The peak hip extension moment (Figure 2d) increased with distance (*p* = 0.001 and *η* ^2^_p_ = 0.448) rising from −1.22 ± 0.07 Nm/kg at 0 km to −1.41 ± 0.07 Nm/kg at 10 km (+18.80% ± 3.98% and *p* = 0.001), −1.47 ± 0.07 Nm/kg at 15 km (+26.86% ± 5.87% and *p* = 0.001), and −1.50 ± 0.07 Nm/kg at 20 km (+28.85% ± 5.70% and *p* < 0.001). In contrast, the peak hip abduction moment (Figure 2e) decreased significantly (*p* = 0.019 and *η* ^2^_p_ = 0.302) from 1.92 ± 0.07 Nm/kg at 0 km to 1.80 ± 0.07 Nm/kg at 15 km (−7.00% ± 2.01% and *p* = 0.012). The peak hip external rotation moment (Figure 2f) also declined (*p* = 0.001 and *η* ^2^_p_ = 0.439) dropping from 0.94 ± 0.05 Nm/kg at 0 km to 0.81 ± 0.05 Nm/kg at 5 km (−12.78% ± 3.53% and *p* = 0.001), 0.77 ± 0.04 Nm/kg at 10 km (−15.31% ± 3.92% and *p* < 0.001), 0.78 ± 0.06 Nm/kg at 15 km (−16.39% ± 3.93% and *p* = 0.001), and 0.82 ± 0.05 Nm/kg at 20 km (−10.53% ± 3.78% and *p* = 0.026). For symmetry, the hip abduction moment symmetry angle (Figure 2h) decreased at 5 km from 32.89% ± 0.98% to 30.50% ± 1.04% (−2.39% ± 0.72% points and *p* = 0.030). The hip external rotation moment symmetry angle (Figure 2i) decreased significantly at 5 km (−6.76% ± 1.53% points and *p* < 0.001), 10 km (−7.42 ± 1.45 and *p* < 0.001), and 15 km (−5.05 ± 1.56 and *p* = 0.002). No significant changes were found in the symmetry angle of the hip extension moment (Figure 2g).

**FIGURE 3:**
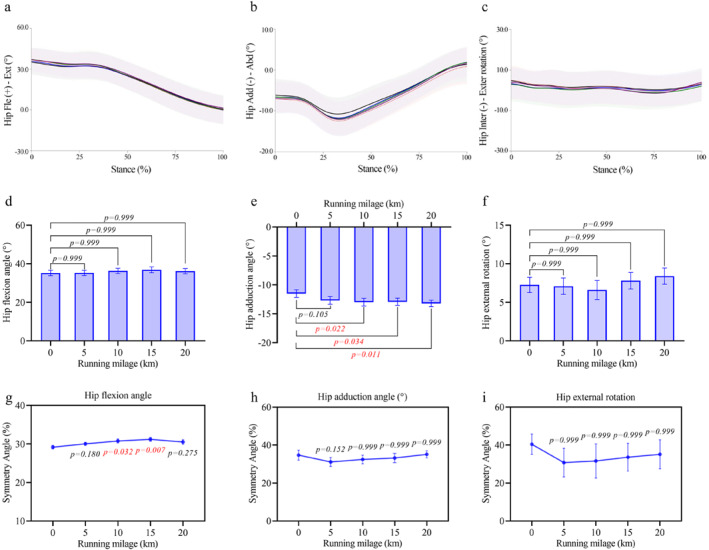
Changes in hip joint angles and their symmetry angles during the half marathon. (a–c) Average angle curves for hip flexion angle (a), hip abduction angle (b), and hip external rotation (c) throughout the stance phase. Shaded areas indicate standard deviation. (d–f) Mean ± SEM values of peak hip flexion angle (d), hip abduction angle (e), and hip external rotation (f) across running distances. (g–i) Mean ± SEM of symmetry angles for hip flexion (g), hip abduction (h), and hip external rotation (i) angles.

Regarding hip joint angles, the peak hip adduction angle (Figure 3e) increased significantly (*p* = 0.014 and *η* ^2^_p_ = 0.316) from −11.50° ± 0.66° at 0 km to −12.99° ± 0.65° at 10 km (+18.36% ± 5.26% and *p* = 0.022), −12.93° ± 0.62° at 15 km (+17.54% ± 5.27% and *p* = 0.034), and −13.20° ± 0.56° at 20 km (+22.48% ± 5.84% and *p* = 0.011). No significant changes were observed in peak hip flexion (Figure 3d) or external rotation angles (Figure 3f). The symmetry angle of hip flexion (Figure 3g) increased significantly at 10 and 15 km (*p* = 0.032 and *p* = 0.007, respectively), whereas symmetry in adduction (Figure 3h) and external rotation (Figure 3i) angles remained unchanged.

### 3.3 Knee Joint

**FIGURE 4:**
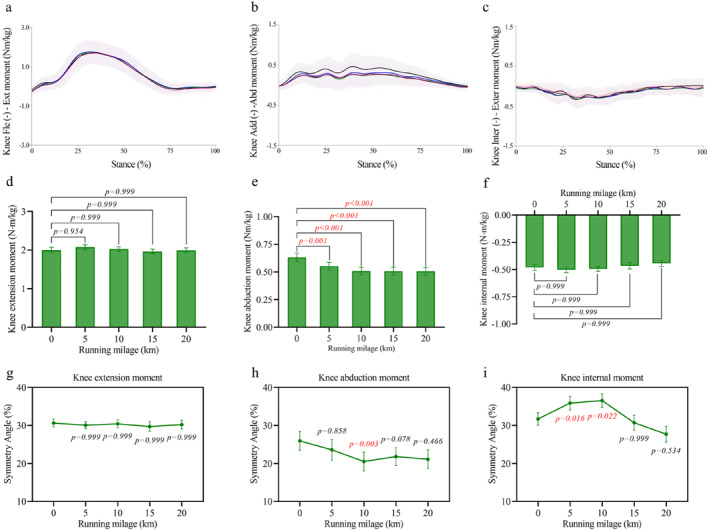
Changes in knee joint moments and their symmetry angles during the half marathon. (a–c) Average moment curves for knee extension (a), knee abduction (b), and knee internal rotation (c) throughout the stance phase. Shaded areas indicate standard deviation. (d–f) Mean ± SEM values of peak knee extension moment (d), knee abduction moment (e), and knee internal rotation moment (f) across running distances. (g–i) Mean ± SEM of symmetry angles for knee extension (g), knee abduction (h), and knee internal rotation (i) moments.

For knee joint moments, the peak abduction moment (Figure 4e) decreased significantly (*p* < 0.001 and *η* ^2^_p_ = 0.559) from 0.63 ± 0.04 Nm/kg at 0 km to 0.55 ± 0.04 Nm/kg at 5 km (−11.77% ± 2.52% and *p* = 0.001) and 0.51 ± 0.03 Nm/kg at 10–20 km (−18% to 20% and all *p* < 0.001). No significant changes were observed in knee extension (Figure 4d) or internal rotation moments (Figure 4f). Regarding symmetry, the knee abduction moment symmetry angle (Figure 4h) decreased significantly at 10 km (−5.42% ± 2.52% points and *p* = 0.003). In contrast, the symmetry angle of the internal rotation moment (Figure 4i) increased significantly at 5 km (+4.16% ± 1.22% points and *p* = 0.016) and 10 km (+4.83 ± 1.68 and *p* = 0.022).

**FIGURE 5:**
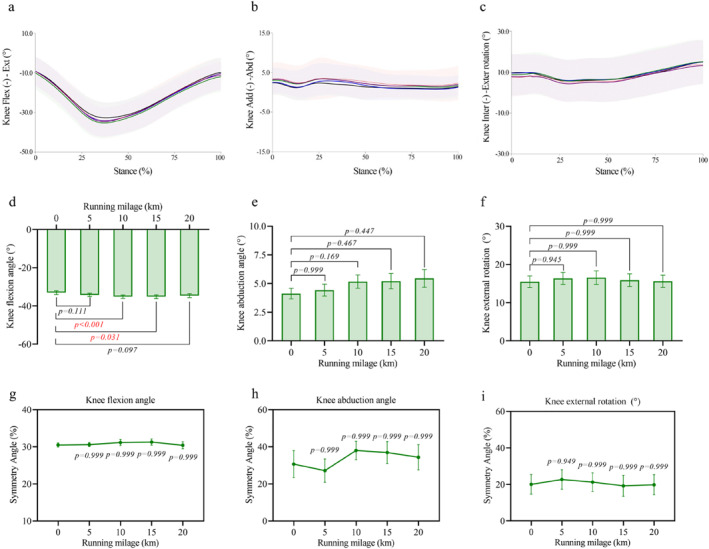
Changes in knee joint angles and their symmetry angles during the half marathon. (a–c) Average angle curves for knee flexion angle (a), knee abduction angle (b), and knee external rotation (c) throughout the stance phase. Shaded areas indicate standard deviation. (d–f) Mean ± SEM values of peak knee flexion angle (d), knee abduction angle (e), and knee external rotation (f) across running distances. (g–i) Mean ± SEM of symmetry angles for knee flexion (g), knee abduction (h), and knee external rotation (i) angles.

For knee joint angles, the peak knee flexion angle (Figure 5d) increased significantly (*p* = 0.002 and *η* ^2^_p_ = 0.393) from −33.00° ± 0.98° at 0 km to −35.12° ± 0.91° at 10 km (+7.22% ± 1.59% and *p* < 0.001) and −35.15° ± 0.97° at 15 km (+7.56% ± 2.26% and *p* = 0.031). No significant changes were found in peak abduction (Figure 5e) or external rotation angles (Figure 5f) nor in any of the corresponding symmetry angles (Figure 5g–i).

## 4 Discussion

This study aimed to clarify the biomechanical adaptations recreational runners experience during a half marathon. Consistent with our hypothesis and previous studies (Renner and Queen 2021; Giovanelli et al. 2017; Gerlach et al. 2005), peak vertical GRF decreased and contact time increased as mileage progressed from 0 to 20 km. The reduction reflects a decreased ability of the runners to generate force. Meanwhile, the increase in contact time may help preserve forward momentum despite reduced force output (Prigent et al. 2022). Impulse showed a decline after 10 km. Although it remained stable in the initial stages, this decrease suggests that the strategy was insufficient to offset the effects of fatigue.

Mileage accumulation also led to distinct adaptations in hip and knee mechanics. In the sagittal plane, the increased hip extension moment likely reflects greater reliance on proximal musculature to compensate for distal fatigue and sustain propulsion (Simpson and Bates 1990). The observed increase in knee flexion angle at 10 and 15 km suggests enhanced energy absorption at the knee, potentially increasing patellofemoral joint loading and elevating the risk of patellofemoral pain (Takabayashi et al. 2019).

In the frontal plane, a reduction in knee abduction moment from 5 km onward may indicate decreased lateral stability, which could heighten injury risk (Chen et al. 2022). The significant increase in hip adduction angle from 10 km is another concern as it is associated with patellofemoral pain syndrome in runners (Patrek et al. 2011). These frontal plane changes may increase loading on medial structures of the hip and knee, contributing to overuse injuries (Neal et al. 2016). In contrast, adaptations in the horizontal plane were less prominent, with only a gradual reduction in hip external rotation observed. Whether these joint‐specific changes are functionally coordinated or reflect independent fatigue responses remains unclear and warrants further study.

Contrary to our initial hypothesis, most biomechanical parameters did not exhibit progressively worsening asymmetry. In fact, only hip flexion angle at 10 and 15 km and knee internal rotation moment at 5 and 10 km showed increased asymmetry. Interestingly, the symmetry angles of ground reaction force, hip abduction moment, and hip external rotation moment exhibited increased symmetry at 5 km. These findings may indicate an early coordination strategy aimed at maintaining lower limb stability under emerging fatigue (Heil et al. 2020). This neuromuscular adjustment could help mitigate unilateral overuse risk during prolonged running (Vagenas and Hoshizaki 1991). Although early changes could also stem from treadmill accommodation or motor pattern optimization (Divert et al. 2005; Morio et al. 2016), all participants had prior treadmill experience and completed a familiarization session, which likely minimized these effects.

Several limitations should be noted. First, the use of a treadmill may not fully replicate outdoor running conditions. Second, the study sample included only recreational runners, which limits generalizability to elite or novice populations with differing fatigue resistance. Future studies should explore how training background influences biomechanical responses to fatigue. Lastly, although not included in this manuscript, additional pre–post strength testing in the same cohort confirmed reductions in lower‐limb torque and power, supporting the presence of neuromuscular fatigue (Wang, Xu, et al. 2025).

## 5 Perspective

This study identified fatigue‐induced adaptations in GRF, ground contact time, impulse, and hip and knee joint mechanics in recreational runners during a half marathon. These adaptations likely reflect compensatory strategies aimed at maintaining locomotor function under increasing fatigue. Notably, fatigue did not consistently increase asymmetry; instead, some parameters became more symmetrical, suggesting an active coordination strategy to preserve interlimb balance. Future studies should investigate the relationship between fatigue‐induced changes in lower‐limb joint symmetry and the development of running‐related injuries.

## Consent

Written informed consent was provided by all participants prior to data collection.

## Conflicts of Interest

The authors declare no conflicts of interest.

## Data Availability

Data are available upon reasonable request.
